# Dose–response association between dietary patterns and gestational diabetes mellitus risk: A systematic review and meta‐analysis of observational studies

**DOI:** 10.1002/fsn3.3042

**Published:** 2022-09-27

**Authors:** Fahimeh Haghighatdoost, Roya Riahi, Shahla Safari, Zahra Heidari

**Affiliations:** ^1^ Isfahan Cardiovascular Research Center Cardiovascular Research Institute, Isfahan University of Medical Sciences Isfahan Iran; ^2^ Department of Biostatistics and Epidemiology School of Health, Isfahan University of Medical Sciences Isfahan Iran; ^3^ Cardiac Rehabilitation Research Center Cardiovascular Research Institute, Isfahan University of Medical Sciences Isfahan Iran

**Keywords:** dietary pattern, gestational diabetes mellitus, meta‐analysis, pregnancy

## Abstract

Although dietary factors are relevant modifiable risk factors for gestational diabetes mellitus (GDM), the exact association between dietary patterns and GDM remains controversial. Therefore, a systematic review and dose–response meta‐analysis of observational studies were conducted to summarize the association between dietary patterns and risk of GDM. PubMed, Scopus, Web of Science, Cochrane Library, and EMbase databases were systematically searched for publications up to March 10, 2020. All observational studies which assessed the risk of GDM according to the categories of healthy or unhealthy dietary patterns derived by either a priori or a posteriori methods were eligible to be included. Pooled effect sizes for the highest vs. lowest categories of healthy/unhealthy dietary patterns were calculated using the random‐effects model. Linear and nonlinear dose–response analyses were performed to determine dose–response associations. Thirty‐one studies were included, of which 26 studies (80,849 participants) assessed healthy dietary pattern and 15 studies (32,965 participants) assessed the unhealthy dietary pattern. Individuals with a higher adherence to the healthy dietary pattern were less likely to be affected by GDM (RR = 0.86; 95% CI: 0.76–0.96; *I* ^2^ = 56.2%). There was a marginally significant association between unhealthy dietary patterns and GDM risk (RR = 1.28; 95% CI: 0.99–1.67; *I* ^2^ = 74.7). Significant linear associations were observed between healthy (*p* = .011) and unhealthy (*p* = .009) dietary patterns and GDM risk. Pregnant women with a healthier dietary pattern (a diet rich in fruits, vegetables, and whole grains) had lower risk for GDM. In contrast, higher adherence to an unhealthy dietary pattern was associated with increased risk of GDM. Further longitudinal studies are needed to confirm the results.

## Graphical abstract

To the best of our knowledge, there is no information regarding the strength and shape of a dose‐response relation between dietary patterns and risk of GDM; therefore, to provide further insights regarding the association between dietary patterns and GDM risk, we conducted a systematic review and dose‐response meta‐analysis to summarize the association between dietary patterns (healthy, unhealthy) and risk of GDM on all available observational studies

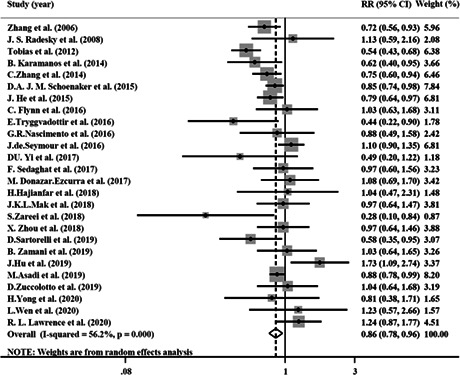

## 1 INTRODUCTION

Gestational diabetes mellitus (GDM) is defined as diabetes diagnosed in the second or third trimester of pregnancy that was not clearly overt diabetes prior to gestation (American Diabetes Association [ADA], 2020). GDM is one of the most common pregnancy complications worldwide with a global prevalence of 14.0% (varying from 7.5% to 27.0% among different areas; Wang et al., 2022). In 2021, the International Diabetes Federation (IDF) estimated that 16.7% of live births were affected by hyperglycemia in pregnancy, among which about 75%–90% was due to GDM (Atlas IDFD, 2021).

GDM has notable adverse effects on the health status of mothers and their offspring during pregnancy, delivery, or even later in life. Risk factors for GDM include several factors, such as maternal age, excess body weight, race, family history of diabetes, and lifestyle (Mirghani Dirar & Doupis, 2017) and the combination of various risk factors can synergistically increase the risk of GDM (Popova et al., 2015). Previous evidence suggested that dietary patterns were associated with the risk of developing GDM (Bao, 2016; Mirghani Dirar & Doupis, 2017; Reader et al., 2006) but findings were not fully consistent (Izadi et al., 2016; Tobias et al., 2012; Tryggvadottir et al., 2016). For example, while some studies have shown that higher adherence to the Western dietary pattern (high in red and processed meat, refined grains, sugar, and fried foods) was associated with an increased risk of GDM (de Seymour et al., 2016; Hassanizadeh et al., 2020; He et al., 2015; Izadi et al., 2016; Schoenaker et al., 2015; Zareei et al., 2018; Zhang & Ning, 2011), some others suggested a null association (de Seymour et al., 2016; He et al., 2015). A similar discrepancy has also been reported for healthy dietary patterns which are highly loaded with fruits, vegetables, low‐fat dairy products, and whole grains (Bao et al., 2014; de Seymour et al., 2016; Flynn et al., 2016).

To date, two meta‐analyses and systematic reviews have been tried to summarize the association between dietary patterns and GDM (Hassanizadeh et al., 2020; Kibret et al., 2019). They showed that greater adherence to Western dietary patterns was significantly and positively associated with the risk of GDM, while healthy dietary patterns were inversely related to the risk of GDM (Hassanizadeh et al., 2020; Kibret et al., 2019). However, Kibret et al.'s study is based on a few effect sizes for dietary patterns (Hassanizadeh et al., 2020), and Hassanizadeh et al.'s study has separately analyzed healthy/unhealthy dietary patterns without combining these patterns (Kibret et al., 2019). In addition, these meta‐analyses missed some eligible publications and focused on cohort studies. Moreover, none of them examined the dose–response association (Kibret et al., 2019; Hassanizadeh et al., 2020).

To the best of our knowledge, there is no information regarding the strength and shape of a dose–response relationship between dietary patterns and risk of GDM. Therefore, a systematic review and dose–response meta‐analysis were conducted to summarize the association between dietary patterns (healthy, unhealthy) and risk of GDM in all available observational studies.

## 2 METHODS

The protocol of this review and meta‐analysis follows the Preferred Reporting Items for Systematic reviews and Meta‐Analyses (PRISMA) statement (Moher et al., 2009).

### 2.1 Search strategy

Table 1 shows the inclusion and exclusion criteria based on the PICOS framework. The search was carried out using PubMed, Scopus, Web of Science, Cochrane Library, and EMbase databases. It was updated until March 10, 2020, using the following keywords: (“diet* pattern*” OR “eating pattern*” OR “food* pattern*” OR “diet* habit*” OR diet* OR “maternal diet* pattern*”)) AND (“Diabetes, Gestational” OR “Gestational Diabetes” OR “Diabetes Mellitus, Gestational” OR “Pregnancy‐Induced Diabetes”). In addition, the references cited in the relevant articles or published reviews were carefully evaluated by a manual search for additional pertinent studies. Where there was a lack of information in the publications, the corresponding authors were contacted to complete data. No date, language, or country restrictions were applied. The Mendeley reference manager (version 1.19.4) was used in the review process.

**TABLE 1:** PICOS criteria for inclusion and exclusion of studies

Parameter	Criteria
Population	Pregnant women
Intervention/exposure	Highest adherence to a healthy/unhealthy dietary pattern
Comparator	Lowest adherence to a healthy/unhealthy dietary pattern
Outcome	Incidence of gestational diabetes mellitus (GDM)
Setting or study design	Observational studies (cohort, case–control & cross‐sectional)

### 2.2 Study selection

Two independent reviewers (R.R. and Z.H.) performed study screening and exclusion procedures. Studies were included based on the following selection criteria: (1) evaluation of either a priori or a posteriori dietary patterns as the exposure of interest; (2) reporting the risk of GDM as the outcome of interest; (3) being an original observational study (e.g., cohort study, case–control study, or cross‐sectional study); (4) reporting the usable risk estimates (e.g., relative risks [RRs], hazard ratios [HRs], or odds ratios [ORs]) or presenting necessary data for estimation; and (5) focusing on the general population of pregnant women. Studies were excluded if they (1) investigated nutrients, foods, or food groups separately or did not meet the definition of healthy and unhealthy dietary patterns in the present study; (2) did not report the risk of GDM as an outcome; (3) were not original research (i.e., letters to the editor, editorial or review articles, or unpublished data); and (4) did not report necessary risk estimates (RRs, HRs, or ORs). When two reviewers disagreed, the selection of studies or data extraction was resolved through discussion and consensus.

### 2.3 Data extraction and quality assessment

In the current systematic review and meta‐analysis, the following information was extracted from the included publications: the author's first name, country, year, study design, mean age or age range of participants, sample size, follow‐up duration for longitudinal studies, instruments used to assess dietary intakes, race/ethnicity, time period to assess dietary intakes, the method used to GDM diagnosis, the method used to define dietary patterns, the identified dietary patterns, main findings (effect and 95% CI), and adjusted confounders. For some studies, the corresponding author was contacted to collect incomplete data. If several dietary patterns were reported in a study, the patterns were included that matched well with the definition in the study. Accordingly, the healthy dietary pattern was defined as a diet with high consumption or factor loading of fruits, vegetables, whole grains, and fish, while the unhealthy dietary pattern was defined as a diet with high consumption or factor loading of red or processed meat, refined grains, sweets, and fast foods. The number and name of dietary patterns varied in different studies, so two of the patterns were selected that were most similar to the definitions in the study and they were labeled as healthy or unhealthy.

The risk of bias for included studies in the meta‐analysis was assessed by the National Institutes of Health (NIH) Quality Assessment Tool (National Institutes of Health). A 14‐item form for cohort studies and a 12‐item version for case–control studies were utilized (National Institutes of Health). A modified version of the NIH form was used to assess the risk of bias in the cross‐sectional studies. This modified tool assesses the quality of cross‐sectional studies based on 11 of 14 items for cohort studies. Two reviewers (R.R. & SH.S.) evaluated the risk of bias independently, and inconsistencies were resolved by a third researcher (Z.H.). The questions on these forms generally included items such as the research question or objective, definition of the study population, participation rate, recruitment criteria, inclusion and exclusion criteria, sample size, exposure assessment, timeframe, exposure levels, outcome assessment, loss to follow‐up, and confounding variables. According to these forms, the maximum scores for cohort, case–control, and cross‐sectional studies are equal to 14, 12, and 11, respectively. Each study was rated as good (75% or more of the total score, i.e., a low risk of bias), fair (more than 50% but less than 75% of the total score i.e., moderate risk of bias), or poor (50% or less of the total score, i.e., high risk of bias).

### 2.4 Statistical analysis

Since the incidence and prevalence of GDM are relatively low (<10%) (Behboudi‐Gandevani et al., 2019; Marí‐Sanchis et al., 2018), all effect sizes in the current meta‐analysis were regarded equivalent and pooled as one effect size (i.e., RR). The RR and 95% CIs of the highest level of a healthy/unhealthy dietary pattern compared to the lowest level of a healthy/unhealthy dietary pattern were extracted. To evaluate the association between healthy/unhealthy dietary patterns and the risk of GDM, the pooled RR and 95% CIs were estimated by the random‐effects model. To evaluate heterogeneity between studies, the Cochran *Q* test and *I* ^2^ statistic were employed (Higgins et al., 2003). An *I* ^2^ value equal to or above 75% was regarded as an indication of substantial heterogeneity (Higgins et al., 2003). Subgroup analysis was then performed based on the study design (cohort, cross‐sectional, and case–control), geographical region of the study (Western countries/non‐Western countries), dietary tool (FFQ, dietary recall, and dietary record), energy adjustment (yes/no), the method used to define dietary pattern (a posteriori and a priori), and the time period of dietary intake evaluation (pre‐pregnancy or during the pregnancy). A leave‐one‐out sensitivity analysis was carried out to explore the extent to which a particular study might have affected the result. Potential publication bias was assessed by Begg and Egger's tests (Egger et al., 2008; Sutton et al., 2000). A dose–response meta‐analysis was used to estimate the trend of GDM risk across categories of healthy/unhealthy dietary patterns using the proposed method by Xu and Doi (Xu & Doi, 2018). The parameters of this model are estimated using the inverse variance weighted least squares regression with cluster robust error variances (REMR model), and this model does not require any information in terms of the correlation structure of regression coefficients (Xu & Doi, 2018). A dose–response model with a quadratic trend and also restricted cubic splines with three knots were used to examine a possible nonlinear association between dietary patterns and GDM risk. In the modeling process, the departure from a linear model was also tested. Eighteen studies (Asadi et al., 2019; Donazar‐Ezcurra et al., 2017; Du et al., 2017; He et al., 2015; Hu et al., 2019; Mak et al., 2018; Nascimento et al., 2016; Radesky et al., 2008; Sartorelli et al., 2019; Schoenaker et al., 2015; Tobias et al., 2012; Wen et al., 2020; Yong et al., 2020; Zamani et al., 2019; Zareei et al., 2018; Zhang et al., 2006; Zhou et al., 2018; Zuccolotto et al., 2019) and 12 studies (Asadi et al., 2019; Donazar‐Ezcurra et al., 2017; Hu et al., 2019; Nascimento et al., 2016; Radesky et al., 2008; Sartorelli et al., 2019; Schoenaker et al., 2015; Shin et al., 2015; Yong et al., 2020; Zamani et al., 2019; Zareei et al., 2018; Zhang et al., 2006) with necessary observations were ultimately used for dose–response meta‐analysis of healthy and unhealthy dietary patterns, respectively. Theoretical rationale and codes written to implement this model can be found in the work of Xu and Doi (Xu & Doi, 2018). In the current study, *p* < .05 was considered statistically significant. All statistical analyses were carried out using Stata 11.2 software (StataCorp).

## 3 RESULTS

### 3.1 Search results

**FIGURE 1:**
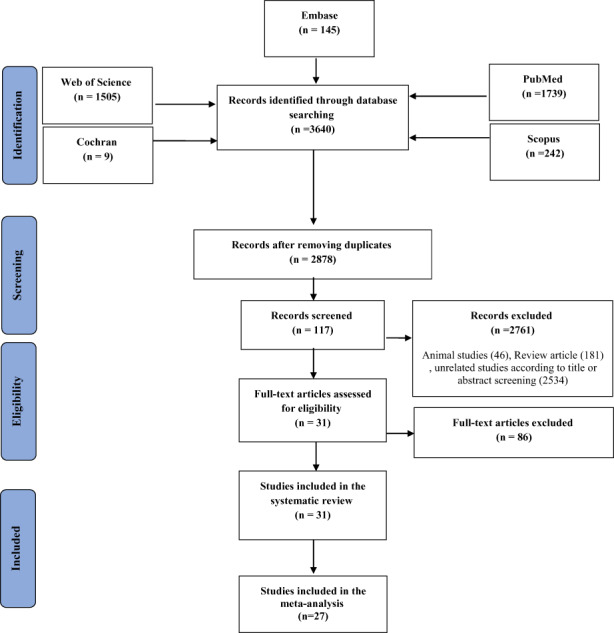
Flowchart of study selection for systematic review and meta‐analysis

The study selection process is represented in Figure 1. According to this flowchart, 2878 citations were retrieved from PubMed, Web of science, Scopus, Embase, and Cochran databases (excluding duplicates, *n* = 762). After screening for titles and abstracts of the identified publications, 2761 articles were excluded, and through the further review using the full texts, 86 additional articles were excluded. Finally, 31 observational studies comprising 18 prospective cohort studies (Bao et al., 2014; Donazar‐Ezcurra et al., 2017; Du et al., 2017; He et al., 2015, 2019; Jarman et al., 2018; Karamanos et al., 2014; Lawrence et al., 2020; Mak et al., 2018; Nascimento et al., 2016; Radesky et al., 2008; Schoenaker et al., 2015; Tobias et al., 2012; Tryggvadottir et al., 2016; Wen et al., 2020; Yong et al., 2020; Zhang et al., 2006, 2014), 7 cross‐sectional studies (de Seymour et al., 2016; Flynn et al., 2016; Hajianfar et al., 2018; Sartorelli et al., 2019; Shin et al., 2015; Zhou et al., 2018; Zuccolotto et al., 2019), and 6 case–control studies (Asadi et al., 2019; Chen et al., 2019; Izadi et al., 2016; Sedaghat et al., 2017; Zamani et al., 2019; Zareei et al., 2018), published between 2006 and 2020, were included in the systematic review (*n* = 31) and meta‐analysis (*n* = 27) (Table 2).

**TABLE 2:** Summary of main characteristics of included studies in the systematic review

ID	Author name	Country, year	Study design	Age (years)	Sample size (*n*)	Follow‐up duration (years)	Dietary assessment tool	Race/ethnicity	Time to dietary pattern assessment	Method used to define GDM diagnosis tool	Method used to define dietary patterns (score)	Dietary patterns identified or dietary scores used	Main findings (effect and 95%CI)	Adjustment for confounders	Score
1	Zhang et al.	USA, 2006	Cohort	24–44	13,110	8	133‐food item semiquantitative FFQ	White, African American, Hispanic, Asian, and others	Prepregnancy	Self‐reported (biennial questionnaire)	Factor analysis	*Prudent pattern*: High intakes of fruit, green leafy vegetables, poultry, and fish. *Western pattern*: High intakes of red meat, processed meat, refined grain products, sweets, French fries, and pizza	0.72 (0.56–0.93) 1.63 (1.20–2.21)	Age, BMI, smoking status race/ethnicity, family history of diabetes, physical activity, and dietary variables including total fat (% energy), cereal fiber, alcohol consumption, glycemic load, and total energy intake	11 (Good)
2	Radesky et al.	USA, 2008	Cohort	32.2	1733	Not reported	FFQ	White Black Hispanic Other	During pregnancy	Non‐fasting oral glucose challenge test	Factor analysis	*Prudent pattern*: High intakes of fruits, tomatoes, cabbages, green leafy vegetables, dark yellow vegetables, legumes, other vegetables, poultry, and fish. *Western pattern*: High intakes of red meat, processed meat, refined grains, snacks, sweets, and desserts, French fries, and pizza	1.13 (0.59–2.16) 0.87 (0.41–1.83)	Maternal age, pregnancy BMI, race/ethnicity, family history of DM, history of GDM in a prior pregnancy, and smoking in the index pregnancy	11 (Good)
3	Tobias et al.	USA, 2012	Cohort	24–44	21,376	10	FFQ	White, African American, Hispanic, Asian and others	Prepregnancy	Self‐report on each biennial questionnaire	aMED (0–8), DASH (8–39), and aHEI (2.5–87.5)	*a MED*: High intakes of Fruit, vegetables, nuts, legumes, soy, fish and seafood, whole grains, moderate alcohol, MUFA:SFA ratio; low intakes of red and processed meats. *DASH*: High intakes of Fruit, vegetables, nuts, legumes, soy, whole grains, low‐fat dairy; low intakes of red and processed meats, sweetened beverages, and Sodium. *a HEI*: High intakes of Fruit, vegetables, nuts, legumes, soy, white: red meat ratio, cereal fiber, moderate alcohol, PUFA:SFA ratio, and multivitamin use; low intake of trans fat	0.76 (0.60–0.95) 0.66 (0.53–82) 0.54 (0.43–0.68)	Age, total energy intake, gravidity, smoking status, PA, parental history of type 2 DM, pre pregnancy BMI, alcohol	10 (Fair)
4	Karamanos et al.	(in 10 Mediterranean countries), 2014	cohort	29	1076	7 months	FFQ	Mediterranean countries	During pregnancy	According to American Diabetes Association (ADA) 2010 guidelines	MED score	MED score based onbread, cereals, legumes, vegetables, fruits, meat, fish, eggs, the ratio of olive oil to animal fat, potatoes, cheese and dairy products	0.62 (0.40–0.95)	Energy intake and body weight gain during pregnancy	12 (Good)
5	Zhang et al.	USA, 2014	Cohort	22–44	14,437	10	FFQ	White Non‐white	Pre pregnancy	Self‐report (biennial questionnaire 2001)	HEI (2.5–77.5)	*HEI*: High intakes of vegetables, fruit, nuts, whole grains, polyunsaturated fatty acids, and long chain omega 3 fatty acids and low intakes of red and processed meats, sugar sweetened beverages, trans fats, and sodium	0.75 (0.59–0.94)	Age, parity, family history of DM, history of infertility, race, EN, alcohol	11 (Good)
6	Bao et al.	USA 2014	Cohort	25–44	21,411	10	Food‐frequency questionnaires	White, African American, Hispanic, Asian, and others	Pre pregnancy	Self‐report on each biennial questionnaire	LCD score	‘Animal LCD score’: a low‐carbohydrate, high animal protein and animal fat dietary pattern and ‘Vegetable LCD score’: a dietary pattern low in carbohydrate and high in vegetable protein and vegetable fat	1.36 (1.13–1.64) 0.84 (0.69–1.03)	Age, parity, race‐ethnicity family history of diabetes, cigarette smoking, alcohol intake, physical activity, total energy intake, BMI	9 (Fair)
7	Schoenaker et al.	Australia, 2015	Cohort	25–30	3858	9	101‐item FFQ	Australian	Pre pregnancy	Self‐reported	Factor analysis	‘Meats, snacks and sweets’ *pattern*: high intakes of red and processed meat, cakes, sweet biscuits, fruit juice, chocolate and pizza. *‘*Mediterranean‐style*’* pattern: High intakes of vegetables, legumes, nuts, tofu, rice, pasta, rye bread, red wine and fish. *‘*Fruit and low‐fat dairy’ *pattern*: high intakes of fruits and low‐fat dairy including yogurt, low‐fat cheese and skimmed milk. ‘Cooked vegetables’ *pattern*: high intakes of carrots, peas, cooked potatoes, cauliflower and pumpkin	1.35 (0.98–1.81) 0.85 (0.76–0.98) 1.01 (0.76–1.37) 1.04 (0.77–1.38)	Age, highest qualification completed, smoking, physical activity, BMI and polycystic ovary syndrome, hypertensive disorders of pregnancy, parity and inter‐pregnancy interval and energy intake	11 (Good)
8	Shin et al.	USA, 2015	Cross‐sectional	16–41	249	—	Interviewer‐administered 24 h recall, and cell phone	Mexican American or other Hispanic Non‐Hispanic white Non‐Hispanic black Other including multi‐racial	During pregnancy	Plasma glucose level > = 5.1 mmol/L	Reduced rank regression	“high refined grains, fats, oils and fruit juice” pattern: high intakes of refined grains, solid fats, oils, and fruit juice. “high nuts, seeds, fat and soybean; low milk and cheese” pattern: high intakes of nuts and seeds, solid fats, soybean products and low intakes of milk and cheese. “high added sugar and organ meats; low fruits, vegetables and seafood” pattern: high intakes of added sugars and organ meats and low intakes of fruits and vegetables and seafood	4.9 (1.4–17.0) 7.5 (1.8–32.3) 22.3 (3.9–127.4)	Age, race/ethnicity, education, family poverty income ratio and marital status; pre pregnancy BMI gestational weight gain energy intake; log‐transformed CRP concentrations	10 (Good)
9	He et al.	China, 2015	Cohort	28.9	3063	2	FFQ	Chinese	During pregnancy	75 g/2 h oral glucose tolerance test	Factor analysis	*Vegetable pattern*: high intakes of root vegetables, beans, mushrooms, melon vegetables, seaweed, other legumes, fruits, leafy and cruciferous vegetables, processed vegetables, nuts, and cooking oil. *Protein‐rich pattern*: high intakes of poultry, red meat, animal organ meat, grains (mainly refined), processed meat, fish, soups, leafy and cruciferous vegetables, and eggs. *Prudent pattern*: high intakes of dairy products, nuts, eggs, fish, soups, fruits, and infrequent intake of processed meat, sugar‐sweetened beverages, and processed vegetables. *Seafood pattern*: high intakes of Cantonese desserts, mollusks and shellfish, and sugar‐sweetened beverages and low intakes of grains (mainly refined) and leafy and cruciferous vegetables	0.79 (0.64–0.97) 0.95 (0.78–1.22) 1 (0.82–1.22) 1.23 (1.02–1.49)	Age, education level, monthly income, pre‐pregnancy BMI, family history of diabetes, parity, and other dietary patterns	11 (Good)
10	Flynn et al.	UK, 2016	Cross‐sectional (Baseline data)	30.5	857	—	101‐item FFQ	Australian	During pregnancy	IADPSG criteria	Factor analysis	‘Fruit and vegetables’ pattern: high intakes of bananas, citrus fruit, dried fruit, fresh fruit, green vegetables, pulses, root vegetables, salad vegetables, tropical fruit and yogurt. ‘African/Caribbean’ *pattern* : high intakes of red meat, cassava, white meat, rice including pilau, fried or jollof rice, plantain and fish. ‘Processed’ pattern: high intakes of chocolate, crisps, green vegetables, potatoes, processed meat and meat products, root vegetables, squash and fizzy drinks, sugar free squash and fizzy drinks and chips. ‘Snacks’ pattern: high intakes of biscuits, cookies, cakes, pastries, chocolate, full fat cheese and sweets	1.03 (0.64–1.68) 2.46 (1.41–4.31) 2.05 (1.23–3.41) 1.24 (0.76–2.01)	Age, parity, ethnicity, BMI, living in a deprived area and treatment allocation	—
11	Tryggvadottir et al.	Island, 2016	Cohort	18–40	168	—	4‐day weighed food record	White, African, American, Hispanic, Asian and others	During pregnancy	2 h, 75 g oral glucose tolerance test	Factor analysis	*Prudent pattern*: Vegetables, Eggs, Vegetable oils, Seafood, Soft drinks, Breakfastcereals, Fruit and berries, Nuts and seed, Pasta/couscous, French fries, Tea, coffee, cocoa powder	0.44 (0.21–0.90)	Age, parity, pre pregnancy weight, energy intake (kcal), weekly weight gain and total MET	11 (Good)
12	Nascimento et al.	Brazil, 2016	Cohort	26.2	838	6 months	FFQ	Brazilian	During pregnancy	Based on criteria developed by the IADPSG, after a 75 g, 2 h OGTT screening	Factor analysis	*Traditional*: high intakes of milk, yogurt, juice, fruit, green leaves, vegetable and fish. *Mixed*: high intakes of cheese, pizza, manioc flour, red meat, and canned food; low intakes of rice, chicken and coffee. *Western*: high intakes of eggs, white bread, savory, pasta, fried food, candies, chocolate, salty snacks and soft drinks.	0.88 (0.49–1.58) 0.93 (0.51–1.71) 0.78 (0.43–1.43)	Age, maternal schooling, monthly income, family history of diabetes, parity, other dietary patterns and body mass index	11 (Good)
13	Seymour et al.	Singapore, 2016	Cross‐sectional	31.5	1247	—	24‐h dietary recall	Chinese Malay Indian	During pregnancy	—	Factor analysis	Vegetable‐fruit rice‐ based‐diet: high intakes of vegetables, fruit, white rice, bread, low‐fat meat and fish, and low intakes of fried potatoes, burgers, carbonated and sugar‐sweetened beverages. *Seafood‐noodle‐based‐diet*: high intakes of soup, fish and seafood products, noodles (flavored and/or in soup), low‐fat meat, and seafood, and low intakes of ethnic bread, legumes and pulses, white rice, and curry‐based gravies. *Pasta‐cheese‐processed‐meat‐diet*: high intakes of pasta, cheese, processed meats, tomato‐based and cream‐based gravies	1.10 (0.90–1.35) 0.74 (0.59–0.93) 0.96 (0.79–1.17)	Age, maternal BMI, energy intake, household income, previous history of GDM, family history of diabetes, ethnicity, education level, birth order, smoking, and alcohol consumption	10 (Good)
14	Izadi et al.	Iran, 2016	Case–control	22–44	460	—	24‐h dietary records	—	During pregnancy	Based on fasting glucose (FG) > 95 mg/dL or 1‐hour post prandial glucose >140 mg/dL for the first time in the pregnancy	DASH and MED score	*DASH diet*: is a low‐glycemic index and low‐ energy dense dietary pattern, which contains high quantities of phytoestrogens, magnesium, potassium and dietary fiber, and *Mediterranean diet (MED diet)*: emphasizing 71 consumption of fruits, vegetables, legumes, whole grains and foods rich in monounsaturated 72 fatty acids (MUFA)	0.20 (0.5–0.7) 0.29 (0.17–0.48)	Age, energy, number of children, socio‐economic status	8 (Fair)
15	Yi et al.	China, 2017	Cohort	28	753	8 months	24‐h dietary recalls	Chinese	During pregnancy	75‐g oral glucose tolerance test	Factor analysis	Western pattern mainly included ‘dairy, baked/fried food and white meat’. Traditional pattern mainly included ‘light‐colored vegetables, fine grain, red meat and tubers’. Mixed pattern mainly included ‘edible fungi, shrimp/shellfish and red meat’. Prudent pattern mainly included ‘dark‐colored vegetables and deep‐sea fish’	1.68 (0.66–4.29) 2.92 (1.19–7.17) 0.70 (0.32–1.55) 0.49 (0.20–1.22)	Age, pre‐pregnancy BMI, education, partner smoking, family history of diabetes, parity, daily food energy intake and physical activity	13 (Good)
16	Sedaghat et al.	Iran, 2017	Case–control	18–40	388	—	147‐item semi quantitative FFQ	Iranian	Pre‐pregnancy	100 g, 3 h oral glucose tolerance test	Factor analysis	*Prudent pattern*: high intakes of liquid oils, legumes, nuts and seeds, fruits and dried fruits, fish and poultry whole, and refined grains. *Western pattern*: high intakes of sweet snacks, jam and tinned fruits, mayonnaise, sugar‐sweetened beverages, salty snacks, solid fats, high‐fat dairy, potatoes, organ meats, eggs, red and processed meat, and tea and coffee, as well as negative factor loading for low fat dairy, legumes, and whole grains	0.97 (0.61–1.56) 1.68 (1.04–2.72)	Pre pregnancy BMI, gestational age, physical activity, family history of diabetes, housing ownership, and building area	9 (Good)
17	Donazar.Ezcurra et al.	Spain, 2017	Cohort	28.75	3455	10.3	FFQ	White	Pre‐pregnancy	Self‐reported (biennial questionnaire)	Factor analysis	*Mediterranean (MDP)*: high intakes of vegetables, fruits, fish, whole grain bread, low‐fat dairy products, nuts, olive oil and poultry. *Western (WDP)*: high intakes of red meat, high‐fat processed meats, potatoes, commercial bakery products, whole dairy products, fast foods, sauces, pre‐cooked foods, eggs, soft drinks and sweets and chocolates	1.08 (0.68–1.70) 1.56 (1.00–2.43)	Age, baseline BMI, family history of diabetes, smoking status, physical activity, previous number of pregnancies and any previous multiple pregnancy	10 (Fair)
18	Hajianfar et al.	Iran, 2018	Cross‐sectional	20–40	812	—	117‐item semi‐quantitative FFQ	Iranian	During pregnancy	Based on fasting plasma glucose concentration more than 95 mg/dL and 1‐h plasma glucose after eating 50 g of glucose, more than 140 mg/dl	Factor analysis	*Western pattern*: high intakes of processed meats, fruits, fruits juice, citrus, nuts, fish, desserts and sweets, sugar, saturated fat, sweet fruit (Melon‐ Persimmon‐ Date‐ Fig‐ Grapes‐ Raisin‐ Berries that have high glycemic index), potato, legumes, coffee, egg, pizza, high fat dairy, whole grain and soft drinks. *Traditional pattern*: high intakes of refined grains, colored vegetables, olive, sugar, salt, spices, unsaturated fat, garlic, onion and tea. *Healthy pattern*: high intakes of green vegetables, leafy vegetables, colored vegetables, fruits, low fat dairy, poultry, bulky vegetables, red meat, citrus, nuts, fish, olive, marinades, sweet fruit, egg and unsaturated fat	0.52 (0.22–1.25) 1.71 (0.75–3.88) 1.04 (0.46–2.31)	Energy intake, age and BMI, socio‐economic status, and physical activity	10 (Good)
19	Mak et al.	China, 2018	Cohort	18–40	1337	From 15 to 20 weeks of gestation until 12 months postpartum	Semi‐quantitative FFQ	Chinese	During pregnancy	Oral glucose tolerance tests	Factor analysis	*Plant‐based pattern*: high intakes of green leafy vegetables, cruciferous vegetables, gourd/melon family vegetables, red or orange vegetables, potatoes, root vegetables, bean vegetables, bean products, mushrooms, fruits, and low intake of lean pork meat. *Meat‐based pattern*: high intakes of pork, pig blood curd, ox tripe, organ meat, processed meat, squid and mushrooms. *‘High protein‐low starch’ pattern*: high intakes of foods rich in protein, including eggs, milk, fish and lean pork meat, and low intakes of noodles and bread	0.97 (0.64–1.47) 0.89 (0.58–1.36) 0.73 (0.48–1.10)	Age, pre‐pregnancy BMI, family history of diabetes, parity, education and total physical activity	10 (Fair)
20	Jarman et al.	Canada, 2018	Cohort	31.4	1545	Not reported	142‐item FFQ	White and not white	During pregnancy	—	Factor analysis	*Healthy pattern*: high intakes of other vegetables, green vegetables, fruit (excluding juice), orange vegetables, oils, brown pasta or rice, fish, tomatoes, and white pasta. “Meat and refined carbohydrates pattern”: high intakes of red meat, processed meat, fries and roast potatoes, white bread, and boiled potatoes. “beans, cheese and salad” pattern: high intakes of beans and pulses, cheese, and salad vegetables. “Tea and coffee pattern”: high intakes of coffee, tea (both regular and decaffeinated), reduced‐fat milk, full‐fat milk, cream and added sugar	Univariate associations between dietary pattern scores and gestational diabetes were not observed	Total energy intake	9 (Fair)
21	Zareei et al.	Iran, 2018	Case–control	Control = 27.91 and case = 30.89	204	—	168 items semi‐quantitative FFQ	Iranian	During pregnancy	Glucose challenge test (GCT)	Factor analysis	*Healthy pattern*: high intakes of leafy green vegetables, fruits, poultry, fish, etc. *Unhealthy pattern*: high intakes of mayonnaise, soda, pizza, sugar, etc.	0.28 (0.10–0.84) 2.84 (1.04–7.75)	Age, educational level, BMI weight changes in pregnancy, Number of deliveries, Getting Gestational Diabetes in previous pregnancy, Job, Physical activity	9 (Good)
22	Zhou et al.	China, 2018	Cross‐sectional	17–45	2755	—	Semi‐quantitative FFQ	Han Chinese others	During pregnancy	Based on the results of a 75‐g, 2‐h oral glucose tolerance test	Factor analysis	Beans–vegetables: high intakes of root vegetables, melons and solanaceous vegetables, mushrooms and algae, beans and bean products (soyabean, mung bean, soyabean milk), and leafy and cruciferous vegetables. *Nuts–whole grains*: high intakes of nuts, whole grains and dairy products (milk, milk powder and yogurt). *Organs–poultry– seafood*: high intakes of animal organs, blood, seafood and poultry. *Fish–meat–eggs*: high intakes of freshwater fishes, red meat and eggs. *Rice–wheat–fruits*: high intakes of rice, wheat products and fruits	0.97 (0.64–1.46) 1.25 (0.84–1.86) 1.01 (0.68–1.51) 1.83 (1.21–2.79) 0.72 (0.48–1.08)	Maternal age, ethnology, maternal education, average personal income, family history of diabetes, family history of obesity, smoking, alcohol, parity, pre‐pregnancy BMI, macronutrients, weight gain before GDM diagnosis, total energy intake, other nutrients, and each food or food group component of each dietary pattern.	9 (Good)
23	Sartorelli et al.	Brazil, 2019	Cross‐sectional	≥20	785	—	24‐h dietary recalls	Brazilian	During pregnancy	Based on the WHO criteria	Reduced rank regression	Dietary pattern 1: High intakes of rice, beans, vegetables with low full‐fat dairy, biscuits, and sweets. Dietary pattern 2: High intakes of red meats, full‐fat dairy, chocolate powder and fruits with low chicken and margarine	0.58 (0.36–0.95) 1.48 (0.91–2.40)	Age, education, smoking habits, physical activity, parity, prior GDM, family history of GDM, excess body weight, total energy intake, and dietary underreporting	9 (Good)
24	Zamani et al.	Iran, 2019	Case–control	22–44	460	—	Three 24‐h dietary records	Iranian	During pregnancy	Based on fasting blood glucose >5.27 mmol/L or 1‐hour postprandial glucose >7.77 mmol/L	PDI, hPDI and uPDI score (18–180)	Healthy plant‐based diet (hPDI) included whole grains, fruits, vegetables, nuts, legumes, vegetable oils and tea/coffee. Unhealthy plant‐based diet index (uPDI) included fruit juices, sugar‐sweetened beverages, refined grains, potatoes and sweets/desserts were considered as unhealthy plant foods	1.03 (0.64–1.65) 1.65 (0.98–2.78)	Age and energy intake smoking status, number of children and BMI	8 (Fair)
25	Hu et al.	China, 2019	Cohort	30.32	1014	Not reported	Three‐day food diaries (TFD) and FFQ	Han and Minority	During pregnancy	75 g 2‐h oral glucose tolerance test	Factor analysis	“Traditional pattern (TFD)”: high intakes of tubers, vegetables, fruits, rice, red meat, eggs, and nuts. “Sweet foods pattern (TFD)”: high intakes of pastry and candy, sweet beverages, shrimps, crabs, mussels, fruits, and red meat. “Fried food–beans pattern (TFD)”: high intakes of fried foods, beans and products, and dairy products, and a low intake of organ meats. “Whole grain‐seafood pattern (TFD)”: high intakes of whole grains, shrimps, crabs, mussels, nuts, and seaweed, and a low intake of eggs, dairy products, and rice	0.40 (0.23–0.71) 0.73 (0.46–1.16) 1.08 (0.67–1.74) 1.73 (1.10–2.74)	Other dietary patterns derived from the same dietary assessment tool (TFD or FFQ); pre‐pregnancy BMI, age, parity, family income, education level, ethnicity, smoking status, total energy intake, and physical activity	11 (Good)
26	Asadi et al.	Iran, 2019	Case–control	GDM: 29; Control: 27.5	178	—	67‐item validated FFQ	Iranian	during pregnancy	75 g oral glucose tolerance test (OGTT)	Factor analysis	**Prudent pattern**: high intakes of fruits, low‐fat dairy, potato, egg, fish, poultry, nuts, organs meat and red meat. **Western pattern**: high intakes of sugar‐sweetened beverages, refined grain products, fast foods, salty snacks, sweets and biscuit, mayonnaise and saturated oils	0.88 (0.44–0.99) 1.50 (0.74–3.03)	Pre‐pregnancy BMI, educational Level, occupational situation, history of fetal macrosomia, daily calorie intake, age, and physical Total energy intake, maternal age, high blood pressure, education, maternal BMI, parity, and family history of diabetes	9 (Good)
27	Zuccolotto et al.	Brazil, 2019	Cross‐sectional	27.6 (5.4)	785	—	Two 24‐h dietary recalls	—	During pregnancy	World Health Organization criteria of 2014	Factor analysis	“Traditional Brazilian” pattern: high intakes of rice; bean; meat; vegetables, and low intakes of hard and soft cheese; snacks pizzas and sandwiches. “Snacks” patterns: high intakes of breads; butter and margarine; milk and yogurt; hard and soft cheese; sweets; chocolate milk and cappuccino. “Coffee” pattern: high intakes of coffee; sugar, butter and margarine. “Healthy” pattern: high intakes of vegetables; fruit and natural fruit juice; and low intakes of soda and artificial juice.	0.64 (0.39–1.04) 0.96 (0.59–1.55) 0.97 (0.59–1.59) 1.04 (0.64–1.68)	Age, gestational week at the time of the interview, previous GDM, schooling,, family history of DM, smoking, physical activity and number of children, maternal excessive body weight	11 (Good)
28	Chen et al.	China, 2019	Case–control	—	9556	—	33 food item semi‐quantitative	—	Early pregnancy	75 g oral glucose tolerance test	Factor analysis	*The* “vitamin” nutrient patterns: the most contributed nutrients to them were vitamin A, carotene, vitamins B2 and B6, vitamin C, and calcium, followed by potassium, dietary fiber, and folate	0.71 (0.61–0.84) 0.71 (0.60–0.84) 0.74 (0.63–0.87)	Total energy intake, maternal age, high blood pressure, education, maternal BMI, parity, and family history of diabetes	8 (Fair)
29	Yong et al.	Malasia, 2020	Cross‐sectional	30.04 in non‐GDM and 29.80 in GDM women	452	—	126‐food item semi‐quantitative FFQ	Malay and Non‐Malay	During pregnancy	75 g 2‐h oral glucose tolerance test	Factor analysis	DP 4: high intakes of vegetables, nuts, seeds & legumes, green leafy vegetables, fruits. *DP 5*: high intakes of condiments & spices, and sugar, spread & creamer, oils & fats. *DP 6*: high intakes of protein (poultry, meat, processed meat, dairy, egg and fish), sugars (mainly as high energy beverages, and sweet foods), and energy (bread, cereal & cereal products, rice, noodles & pasta)	0.81 (0.38–1.71) 0.28 (0.11–0.68) 1.15 (0.54–2.47)	Age, ethnicity, medical history of GDM, family history of DM, clinic, gestational week at OGTT performed, maternal age, ethnicity, medical history of GDM and family history of DM.	12 (Good)
30	Wen et al.	China, 2020	Cohort	29.6	324	3	93‐item FFQ	Han Chinese and others	During pregnancy	75 g 2‐h oral glucose tolerance test	Factor analysis	“Vegetable‐based pattern”: high intakes of root vegetables, gourd/ melon family vegetables, freshwater fish, leafy and cruciferous vegetables, and red meat. “Poultry‐and‐fruit‐based pattern”: high intakes of poultry, fresh fruit, processed fruit, soups and meat innards. “Sweet‐based pattern”: high intakes of biscuits, pastries, cakes, bread and deep‐sea fish and seafood products. “Plant‐protein‐based pattern”: high intakes of soya milk, legumes, beans or bean products, buns and rice	1.23 (0.57–2.66) 0.96 (0.45–2.03) 1.97 (0.94–4.12) 1.02 (0.49–2.09)	Age, ethnicity, pre‐pregnancy BMI, education level, smoking status, parity, previous history of GDM, family history of diabetes mellitus (DM) and other dietary pattern	11 (Good)
31	Lawrence et al.	New Zealand, 2020	Cohort	32.32	5384	Not reported	44‐item FFQ	European, Māori, Pacific, Asian, and Other	During pregnancy	Fasting plasma glucose of ≥5.5 mmol/L or a 2 h plasma glucose ≥9.0 mmol/L post 75 g oral glucose tolerance test (OGTT)	Factor analysis	‘Junk’ pattern: high intakes of confectionary, snacks, takeaways, hot chips, processed meats, soft and energy drinks, battered fried fish or seafood, ice‐cream and cakes or biscuits. ‘Health conscious’ *pattern*: high intakes of vegetables, cheese, brown wholemeal bread, non‐citrus fruits, yogurt, dried fruits, high fiber cereal, and Vegemite™ or Marmite™. ‘Traditional/White bread’ pattern: high intakes of whole or standard milk, white bread, margarine, jam honey marmalade, peanut butter, Nutella™ and low fiber and/or high sugar cereals. ‘Fusion/Protein’ pattern: high intakes of noodles, rice, pasta, seafood, chicken, green leafy vegetables, eggs and red meat	0.49 (0.34, 0.70) 1.24 (0.87, 1.77) 0.47 (0.32, 0.68) 1.25 (0.87, 1.81)	Age, ethnicity,NZDep06 score, pre‐pregnancy BMI, pre‐pregnancy and first trimester physical activity, smoking pattern, alcohol consumption and dietary pattern score	10 (Fair)

### 3.2 Description of studies and narrative review

Thirteen studies were conducted in western countries (Donazar‐Ezcurra et al., 2017; Flynn et al., 2016; Karamanos et al., 2014; Nascimento et al., 2016; Radesky et al., 2008; Sartorelli et al., 2019; Schoenaker et al., 2015; Shin et al., 2015; Tobias et al., 2012; Tryggvadottir et al., 2016; Zhang et al., 2006, 2014; Zuccolotto et al., 2019) and 14 studies in non‐Western countries (Asadi et al., 2019; de Seymour et al., 2016; Du et al., 2017; Hajianfar et al., 2018; He et al., 2015; Hu et al., 2019; Lawrence et al., 2020; Mak et al., 2018; Sedaghat et al., 2017; Wen et al., 2020; Yong et al., 2020; Zamani et al., 2019; Zareei et al., 2018; Zhou et al., 2018). The sample size of the studies ranged from 168 to 21,411 in cohort studies, from 249 to 2755 in cross‐sectional studies, and from 178 to 9556 in case–control studies. Participants aged 16 years and over. Twenty‐three studies assessed dietary intake using a validated food frequency questionnaire (FFQ) (Asadi et al., 2019; Bao et al., 2014; Chen et al., 2019; Donazar‐Ezcurra et al., 2017; Flynn et al., 2016; Hajianfar et al., 2018; He et al., 2015; Hu et al., 2019; Jarman et al., 2018; Karamanos et al., 2014; Lawrence et al., 2020; Mak et al., 2018; Nascimento et al., 2016; Radesky et al., 2008; Schoenaker et al., 2015; Sedaghat et al., 2017; Tobias et al., 2012; Wen et al., 2020; Yong et al., 2020; Zareei et al., 2018; Zhang et al., 2006, 2014; Zhou et al., 2018), and eight studies used dietary recall or record (de Seymour et al., 2016; Du et al., 2017; Izadi et al., 2016; Sartorelli et al., 2019; Shin et al., 2015; Tryggvadottir et al., 2016; Zamani et al., 2019; Zuccolotto et al., 2019). Dietary patterns were defined using a posteriori method in 25 studies (Asadi et al., 2019; Chen et al., 2019; de Seymour et al., 2016; Donazar‐Ezcurra et al., 2017; Du et al., 2017; Flynn et al., 2016; Hajianfar et al., 2018; He et al., 2015; Hu et al., 2019; Jarman et al., 2018; Lawrence et al., 2020; Mak et al., 2018; Nascimento et al., 2016; Radesky et al., 2008; Sartorelli et al., 2019; Schoenaker et al., 2015; Sedaghat et al., 2017; Shin et al., 2015; Tryggvadottir et al., 2016; Wen et al., 2020; Yong et al., 2020; Zareei et al., 2018; Zhang et al., 2006; Zhou et al., 2018; Zuccolotto et al., 2019) and a priori method in six studies (Bao et al., 2014; D.K. et al. 2012; Izadi et al., 2016; Karamanos et al., 2014; Zamani et al., 2019; Zhang et al., 2014). Most studies diagnosed GDM according to valid instructions such as the American Diabetes Association (ADA) guidelines and based on glucose challenge test (GCT), glucose tolerance test (GTT), and oral glucose tolerance test (OGTT) (*n* = 24; Asadi et al., 2019; Chen et al., 2019; de Seymour et al., 2016; Du et al., 2017; Flynn et al., 2016; Hajianfar et al., 2018; He et al., 2015; Hu et al., 2019; Izadi et al., 2016; Karamanos et al., 2014; Lawrence et al., 2020; Mak et al., 2018; Nascimento et al., 2016; Radesky et al., 2008; Sartorelli et al., 2019; Sedaghat et al., 2017; Shin et al., 2015; Tryggvadottir et al., 2016; Wen et al., 2020; Yong et al., 2020; Zamani et al., 2019; Zareei et al., 2018; Zhou et al., 2018; Zuccolotto et al., 2019), and seven studies assessed it as self‐report (Bao et al., 2014; Donazar‐Ezcurra et al., 2017; Jarman et al., 2018; Schoenaker et al., 2015; Tobias et al., 2012; Zhang et al., 2006, 2014). Energy adjustment was performed by 21 studies (Asadi et al., 2019; Bao et al., 2014; Chen et al., 2019; de Seymour et al., 2016; Du et al., 2017; Hajianfar et al., 2018; Hu et al., 2019; Izadi et al., 2016; Jarman et al., 2018; Karamanos et al., 2014; Mak et al., 2018; Radesky et al., 2008; Sartorelli et al., 2019; Schoenakeret al., 2015; Shin et al., 2015; Tobias et al., 2012; Tryggvadottir et al., 2016; Zamani et al., 2019; Zhang et al., 2006, 2014; Zhou et al., 2018). Twenty‐five studies examined the dietary intake of participants during pregnancy, while others examined prepregnancy dietary intake (Donazar‐Ezcurra et al., 2017; Schoenaker et al., 2015; Sedaghat et al., 2017; Tobias et al., 2012; Zhang et al., 2006, 2014). In most studies, the risk estimates of GDM were adjusted for confounding variables, such as maternal age, smoking status, race–ethnicity, alcohol consumption, family history of diabetes, physical activity, total energy, and body mass index (BMI).

Data from 26 studies were included in the meta‐analysis of the healthy dietary pattern (Asadi et al., 2019; de Seymour et al., 2016; Donazar‐Ezcurra et al., 2017; Du et al., 2017; Flynn et al., 2016; Hajianfar et al., 2018; He et al., 2015; Hu et al., 2019; Karamanos et al., 2014; Lawrence et al., 2020; Mak et al., 2018; Nascimento et al., 2016; Radesky et al., 2008; Sartorelli et al., 2019; Schoenaker et al., 2015; Sedaghat et al., 2017; Tobias et al., 2012; Tryggvadottir et al., 2016; Wen et al., 2020; Yong et al., 2020; Zamani et al., 2019; Zareei et al., 2018; Zhang et al., 2006, 2014; Zhou et al., 2018; Zuccolotto et al., 2019). In 10 studies, an inverse association was revealed between adherence to the healthy dietary pattern and GDM risk (Asadi et al., 2019; He et al., 2015; Karamanos et al., 2014; Sartorelli et al., 2019; Schoenaker et al., 2015; Tobias et al., 2012; Tryggvadottir et al., 2016; Zareei et al., 2018; Zhang et al., 2006, 2014), 15 studies reported no significant associations (de Seymour et al., 2016; Donazar‐Ezcurra et al., 2017; Du et al., 2017; Flynn et al., 2016; Hajianfar et al., 2018; Lawrence et al., 2020; Mak et al., 2018; Nascimento et al., 2016; Radesky et al., 2008; Sedaghat et al., 2017; Wen et al., 2020; Yong et al., 2020; Zamani et al., 2019; Zhou et al., 2018; Zuccolotto et al., 2019), and 1 study (Hu et al., 2019) reported a direct link. Data from 15 studies were included in the meta‐analysis of the unhealthy dietary pattern (Asadi et al., 2019; Donazar‐Ezcurra et al., 2017; Flynn et al., 2016; Hu et al., 2019; Lawrence et al., 2020; Nascimento et al., 2016; Radesky et al., 2008; Sartorelli et al., 2019; Schoenaker et al., 2015; Sedaghat et al., 2017; Shin et al., 2015; Yong et al., 2020; Zamani et al., 2019; Zareei et al., 2018; Zhang et al., 2006). Six of them revealed a direct association (Donazar‐Ezcurra et al., 2017; Schoenaker et al., 2015; Sedaghat et al., 2017; Shin et al., 2015; Zareei et al., 2018; Zhang et al., 2006) while the association in eight studies was not statistically significant (Asadi et al., 2019; Flynn et al., 2016; Hu et al., 2019; Nascimento et al., 2016; Radesky et al., 2008; Sartorelli et al., 2019; Yong et al., 2020; Zamani et al., 2019), and one study suggested an inverse association (Lawrence et al., 2020).

Due to a lack of data or inconsistency between the definitions of healthy/unhealthy dietary patterns and the ones used by some of the identified articles, data from four studies could not be included in the current meta‐analysis (Bao et al., 2014; Chen et al., 2019; Izadi et al., 2016; Jarman et al., 2018). Therefore, these studies were summarized in the narrative review. One study in China examined the association of a vitamin dietary pattern, characterized by a high intake of vitamin A, carotene, vitamin B2, vitamin B6, folate, vitamin C, dietary fiber, protein, calcium, and phosphorus with the risk of GDM and showed a significant inverse association (OR: 0.90, 95%CI: 0.85–0.95) (Chen et al., 2019). A cohort study in the USA assessed the association of a low‐carbohydrate dietary (LCD) pattern with GDM risk and illustrated that individuals with higher scores of LCD (who consumed lower amounts of carbohydrate) had 27% greater risk for GDM (95% CI: 1.06–1.51; Bao et al., 2014). There were two publications from the same population of Iranian women in which the investigators assessed either the DASH and the Mediterranean dietary score (Izadi et al., 2016) or plant‐based diet index (PDI), healthy plant‐based diet index (hPDI), and unhealthy plant‐based diet index (uPDI; Zamani et al., 2019). Therefore, the results of the newest publication were just included (Zamani et al., 2019). Moreover, Jarman et al.'s (2018) study was excluded due to the lack of data. It reported a null association between the identified dietary patterns and GDM. Although we tried to contact the investigators to get more information, no response was received. Finally, Flynn et al.'s (2016) study is a randomized controlled trial where the association between baseline dietary patterns and the risk of GDM was regarded as a cross‐sectional study in our analysis.

### 3.3 Meta‐analysis

#### 3.3.1 Healthy dietary patterns and risk of GDM

**FIGURE 2:**
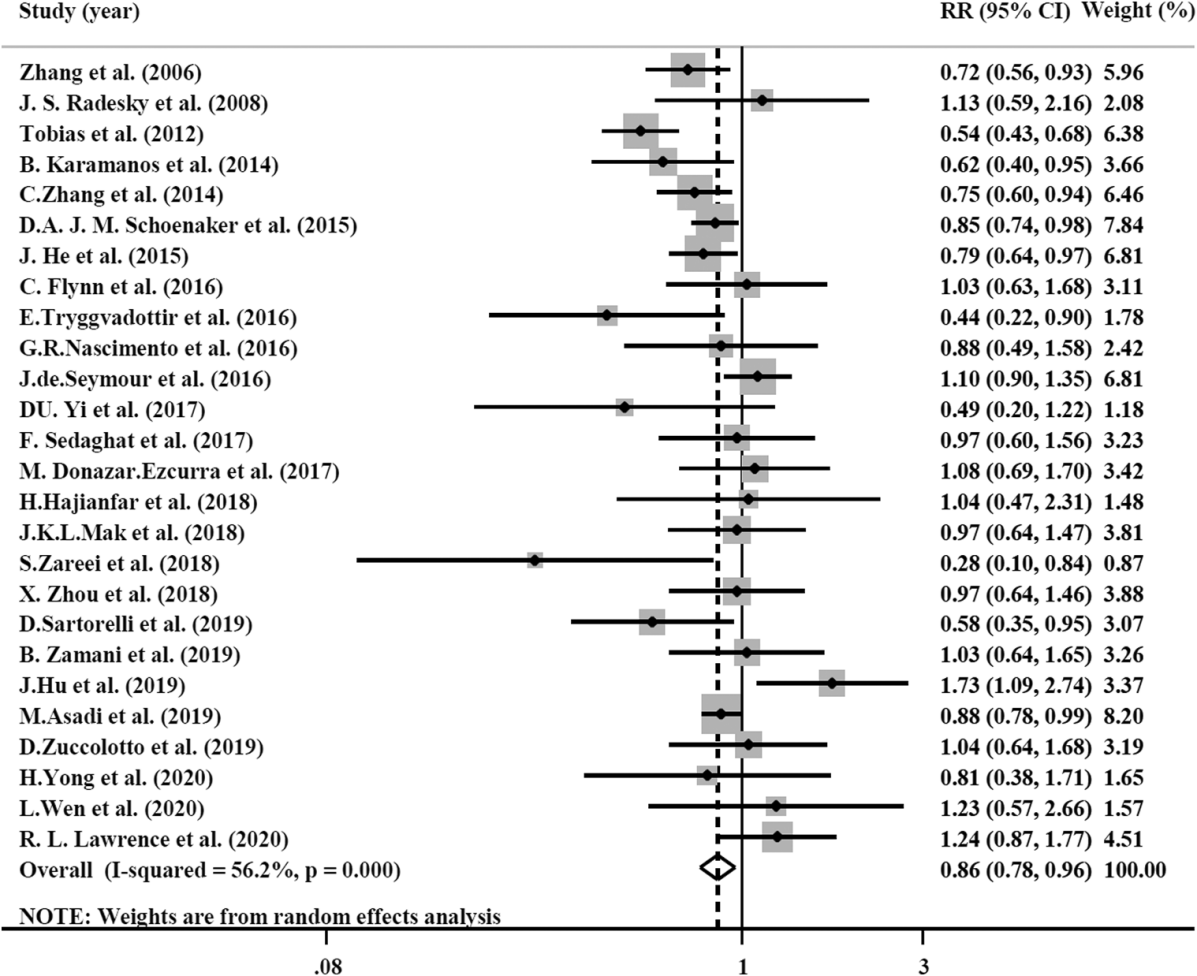
Forest plot of the association between healthy dietary patterns and risk of GDM

The results of the meta‐analysis of the healthy dietary pattern are shown in Figure 2. Individuals with higher adherence to the healthy dietary pattern were less likely to be affected by GDM (RR = 0.86; 95% CI, 0.76–0.96). There was medium heterogeneity between studies (*I* ^2^ = 56.2%; *p* < .001). The results of the subgroup analysis are shown in Table 3. Healthy dietary patterns significantly reduced the risk of GDM in cohort studies (RR = 0.83; 95%CI, 0.72–0.97; *I* ^2^ = 62.1%), studies which evaluated dietary intakes before the pregnancy (RR = 0.77; 95% CI, 0.64–0.92; *I* ^2^ = 65%), studies which were conducted in Western countries (RR = 0.76; 95%CI, 0.66–0.88; *I* ^2^ = 50.9%), and studies that used an FFQ as a dietary assessment tool, used an a priori method to identify dietary patterns, and adjusted for energy. No significant association was observed in cross‐sectional (RR: 0.99; 95%CI, 0.84–1.17; *I* ^2^ = 10.4%) and case–control studies (RR: 0.87; 95% CI, 0.68–1.12; *I* ^2^ = 37.3%) as well as the studies which evaluated dietary intakes during the pregnancy (RR = 0.92; 95% CI, 0.81–1.04; *I* ^2^ = 65%). No evidence of publication bias was found using the asymmetry tests for the healthy dietary pattern (Begg's test *p* = .415, Egger's test *p* = .943) (Table 3). The estimated effect size for the effect of healthy dietary patterns on GDM risk was robust in the leave‐one‐out sensitivity analysis.

**TABLE 3:** Summary risk estimates of the associations between dietary patterns and risk of GDM (highest vs. lowest and subgroup analysis).

Factor	Healthy dietary pattern				Unhealthy dietary pattern			
	No. of studies	RR (95% CI)	Heterogeneity		No. of studies	RR (95% CI)	Heterogeneity	
			*I* ^2^ (%)	*p*‐value			*I* ^2^ (%)	*p*‐value
Overall	26	0.862 (0.775, 0.959)	56.2	<.001	15	1.284 (0.985, 1.672)	74.7	<0.001
Subgroup analyses								
Study design								
Cohort	16	0.833 (0.719, 0.965)	62.1	.001	8	1.010 (0.709, 1.435)	80.2	<0.001
Cross‐sectional	6	0.990 (0.835, 1.173)	10.4	.349	3	2.162 (0.894, 5.230)	79.6	0.007
Case–control	4	0.874 (0.680, 1.125)	37.3	.188	4	1.722 (1.268, 2.340)	0	0.775
Dietary tool								
FFQ	20	0.863 (0.77, 0.967)	55.3	.002	12	1.163 (0.883, 1.533)	74.1	<0.001
Dietary recall	4	0.842 (0.58, 1.218)	61.7	.049	2	5.022 (0.356, 70.74)	88.4	0.003
Dietary record	2	0.704 (0.31, 1.612)	73.6	.052	1	1.650 (0.949, 2.870)	—	—
Energy adjustment								
Adjusted	16	0.827 (0.722, 0.947)	66.3	<.001	8	1.394 (1.025, 1.894)	65.4	0.005
Nonadjusted	10	0.940 (0.800, 1.104)	18.3	0.275	7	1.163 (0.742, 1.823)	79.7	<0.001
Method used to define dietary pattern								
A posteriori	22	0.911 (0.822, 1.010)	41.1	.024	14	1.263 (0.955, 1.67)	76.0	<0.001
A priori	4	0.688 (0.535, 0.884)	60.8	.054	1	1.650 (0.949, 2.87)	—	—
Time period of dietary intakes evaluation								
Prepregnancy	6	0.765 (0.640, 0.916)	65	.014	4	1.531 (1.281, 1.83)	0	0.855
During pregnancy	20	0.916 (0.807, 1.038)	45	.016	11	1.210 (0.830, 1.76)	76.4	<0.001
Geographical region of the study								
Non‐Western	14	0.972 (0.850, 1.112)	43.1	.044	7	1.182 (0.733, 1.905)	80.3	<0.001
Western	12	0.760 (0.657, 0.879)	50.9	.021	8	1.384 (1.062, 1.803)	57.1	0.022

Abbreviations: FFQ, food frequency questionnaire; GDM, gestational diabetes mellitus; RR, relative risk.

#### 3.3.2 Unhealthy dietary patterns and risk of GDM

**FIGURE 3:**
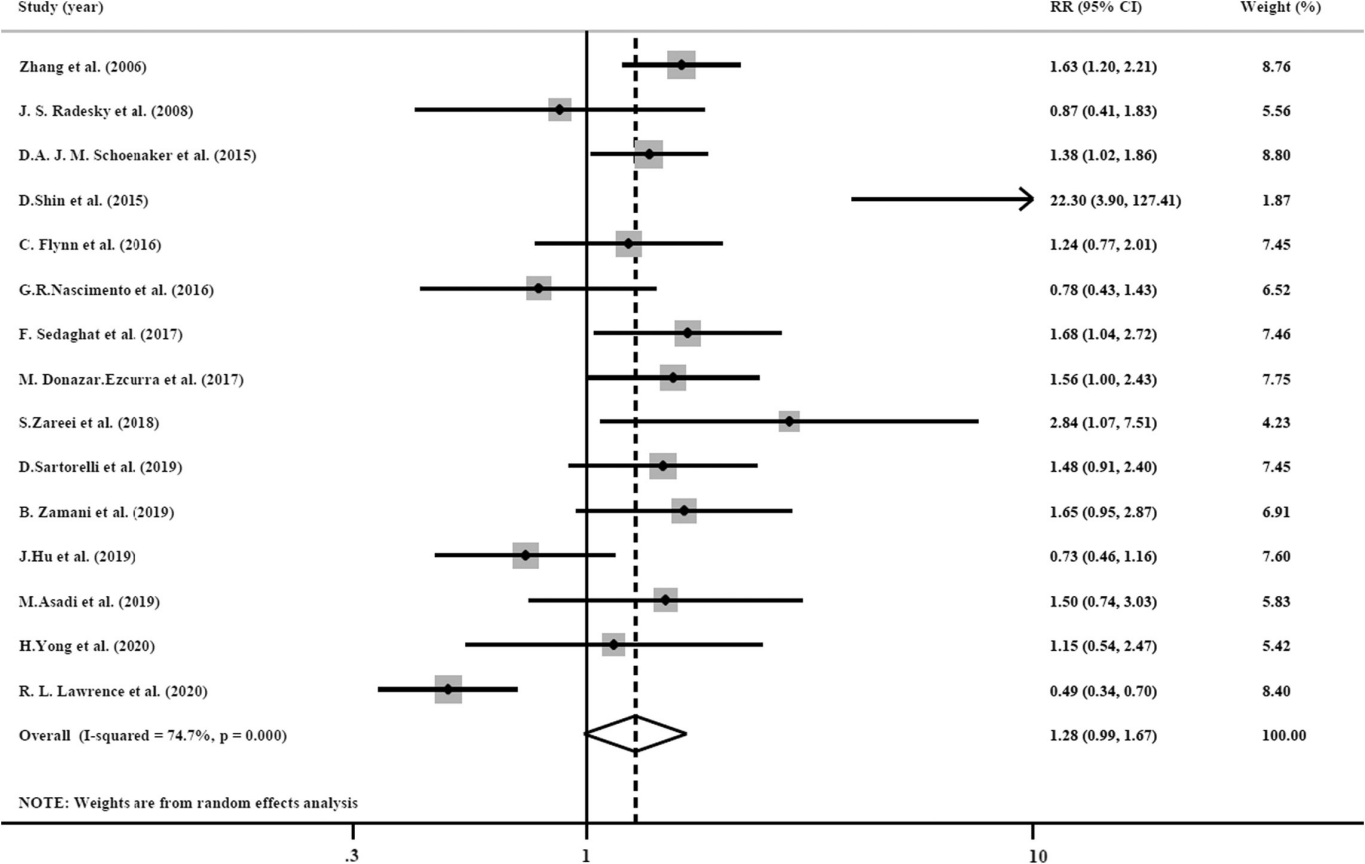
Forest plot of the association between unhealthy dietary patterns and risk of GDM

Results of the meta‐analysis on the association between an unhealthy dietary pattern and GDM risk are shown in Figure 3. The results revealed a marginally significant association between unhealthy dietary patterns and GDM risk (RR = 1.28; 95% CI: 0.99–1.67; *I* ^2^ = 74.7%). The results of subgroup analysis (Table 3) indicated a higher risk for GDM in case–control studies (RR = 1.72; 95% CI: 1.27–2.34; *I* ^2^ = 0%), studies which evaluated prepregnancy dietary intakes (RR = 1.53; 95% CI: 1.28–1.83; *I* ^2^ = 0%), adjusted for energy (RR = 1.39; 95% CI: 1.03, 1.89; *I* ^2^ = 65.4%), and studies which were conducted in western countries (RR = 1.38; 95% CI: 1.06–1.80; *I* ^2^ = 57.1%). No evidence of publication bias was found using the asymmetry tests (Begg's test *p* = .692, Egger's test *p* = .294; Table 3). The results of sensitivity analysis demonstrated that the removal of the studies by Nascimento et al. (2016; RR = 1.33; 95% CI: 1.01–1.75), Hu et al. (2019; RR = 1.34; 95% CI: 1.2–1.77), or Lawrence et al. (2020) (RR = 1.38; 95% CI: 1.12–1.69) led to a significant association between unhealthy dietary pattern and risk of GDM.

#### 3.3.3 Linear and nonlinear dose–response meta‐analysis

**FIGURE 4:**
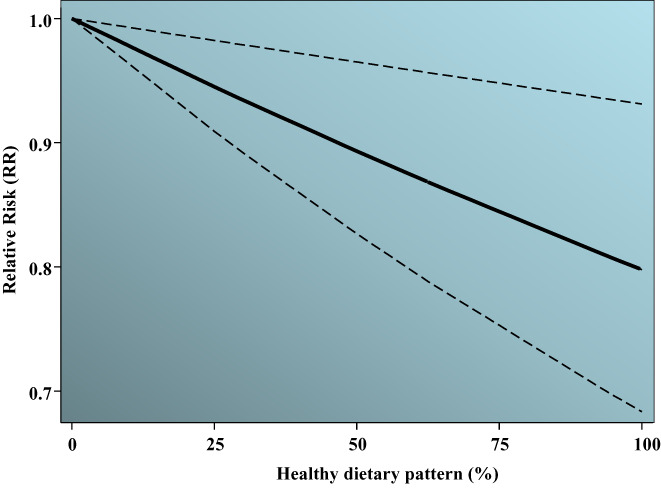
Dose–response plot of the linear relation between the “healthy” dietary pattern and GDM risk

**FIGURE 5:**
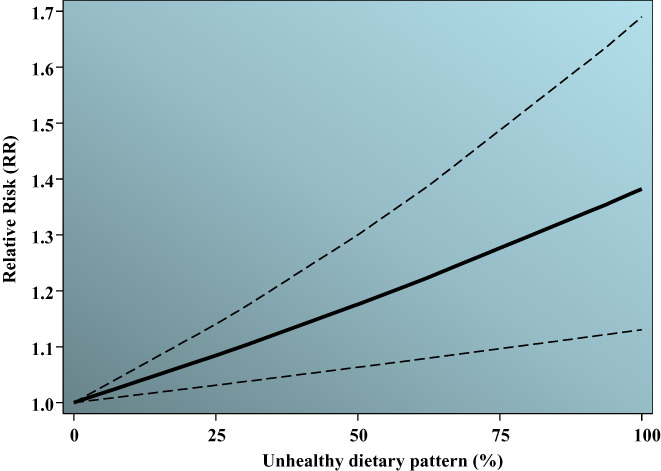
Dose–response plot of the linear relation between the “unhealthy” dietary pattern and GDM risk

Eighteen studies (Asadi et al., 2019; Donazar‐Ezcurra et al., 2017; D. K. et al., 2012; Du et al., 2017; He et al., 2015; Hu et al., 2019; Mak et al., 2018; Nascimento et al., 2016; Radesky et al., 2008; Sartorelli et al., 2019; Schoenaker et al., 2015; Wen et al., 2020; Yong et al., 2020; Zamani et al., 2019; Zareei et al., 2018; Zhang et al., 2006; Zhou et al., 2018; Zuccolotto et al., 2019) and 12 studies (Asadi et al., 2019; Donazar‐Ezcurra et al., 2017; Hu et al., 2019; Nascimento et al., 2016; Radesky et al., 2008; Sartorelli et al., 2019; Schoenaker et al., 2015; Shin et al., 2015; Yong et al., 2020; Zamani et al., 2019; Zareei et al., 2018; Zhang et al., 2006) on the association between healthy and unhealthy dietary patterns with GDM risk were included in dose–response meta‐analysis, respectively. A dataset containing categorically reported risk estimates of GDM and corresponding healthy/unhealthy dietary patterns percentiles was extracted (tertiles [e.g., 33rd and 66th percentiles], quartiles [e.g. 25th, 50th, and 75th], and quantiles [20th, 40th, 60th, 80th]) from the studies. Trends in RRs according to percentile of dietary patterns in each study for the healthy and unhealthy patterns are summarized in Figures S1 and S2, respectively. For the healthy dietary pattern, no significant nonlinear association was found based on the cubic spline model (*p* = .855 for equality of slopes) and quadratic trend (*p* = .993 for equality of slopes; Figure S3). However, a significant linear association was observed between the healthy dietary pattern and GDM risk (*p* = .011). Hence, GDM risk decreased linearly with the percentile of healthy dietary pattern adherence (Figure 4). The model predicted values for 20% and 80% percentiles corresponded to a reduction of 5% (RR in percentile 20th = 0.95, 95% CI: 0.92, 0.97) and 20% (RR in percentile 80th = 0.80 [95% CI: 0.70, 0.92]) in GDM risk, respectively. No significant nonlinear association was observed between the unhealthy dietary pattern and GDM risk based on the cubic spline model (*p* = .249) and quadratic trend (*p* = .121; Figure S4). As shown in Figure 5, a significant linear trend was found (*p* = .009) where GDM risk increased linearly with the percentile of unhealthy dietary pattern adherence. The model predicted values for 20% and 80% percentiles corresponded to 1.10 (95% CI: 1.04, 1.16) and 1.40 (95% CI: 1.15, 1.70), respectively.

#### 3.3.4 Risk of bias

According to the quality assessment tools, all included studies in the meta‐analysis had a low risk of bias (Table 2). Accordingly, the maximum scores for cohort, cross‐sectional, and case–control studies were considered equal to 14, 11, and 12, respectively.

## 4 DISCUSSION

In the current meta‐analysis, available observational studies which evaluated the association between dietary patterns and risk of GDM were systematically reviewed. The findings demonstrated that the highest vs. the lowest adherence to the healthy diet was associated with a decreased risk of GDM, whereas an unhealthy dietary pattern tended to increase the risk of GDM. These associations were dependent on the study design, the instruments used to assess dietary intakes, the methods used to define dietary patterns, adjustment for energy intake, and the geographical region of the study. A statistically significant linear dose–response association was found between both healthy and unhealthy dietary patterns and GDM.

There is a large body of evidence indicating an inverse association between healthful dietary patterns and diabetes risk and a direct link between unhealthy eating patterns and diabetes risk (Fang et al., 2020; Zeraattalab‐Motlagh et al., 2022). However, only a limited number of studies have evaluated the relationship between dietary patterns and GDM. The obtained results in the current meta‐analysis are totally in line with earlier studies which showed that a healthy eating pattern was correlated with a lower risk of GDM (Asadi et al., 2019; Chen et al., 2019; He et al., 2015; Karamanos et al., 2014; Sartorelli et al., 2019; Song et al., 2018; Tobias et al., 2012; Zhang et al., 2006, 2014; Zuccolotto et al., 2019). These findings are also compatible with two earlier meta‐analyses (Hassanizadeh et al., 2020; Kibret et al., 2019). However, one of these studies is based on only 5 and 4 effect sizes for healthy and unhealthy eating patterns (Kibret et al., 2019), respectively, and another one has separately analyzed different dietary patterns without combining these patterns (Hassanizadeh et al., 2020). Moreover, they have missed two eligible cohort publications, and excluded epidemiological studies other than cohort studies. These meta‐analyses also lack subgroup analysis for some relevant confounders. Five further cohort studies (Hu et al., 2019; Lawrence et al., 2020; Mak et al., 2018; Wen et al., 2020; Yong et al., 2020), published after Hassanizadeh et al.'s (2020), were also added to the present meta‐analysis.

Several reasons that may explain these associations through the molecular mechanisms have not been well established. Unhealthy diets are mainly high in red and processed meats. A higher intake of meat, in particular processed red meat, is associated with a higher intake of nitrites which might be converted into nitrosamines (Lijinsky, 1999) in stomach or food products. Nitrosamines are toxic for beta‐cells (Lijinsky, 1999) and potentially can induce type 2 diabetes (Ito et al., 1999). Besides, advanced glycation end products (AGEs), formed through heating and processing in meat and high‐fat products (Peppa et al., 2002), are implicated in the development of diabetes mellitus possibly via their stimulating effect on inflammatory pathways (Vlassara et al., 2002). Red and processed meats are also rich sources of saturated fatty acids and heme iron that may increase oxidative stress and beta‐cell damage (Zhang et al., 2006). A large prospective cohort study demonstrated that adjustment for red and processed meat disappeared the significant adverse association between the Western dietary pattern and GDM risk, while red and processed meat were independently linked to the risk of GDM (Zhang et al., 2006). Another possible explanation might be related to the close and positive correlation between Western dietary patterns and fasting glucose, insulin, and C peptide (Fung et al., 2001). Therefore, prepregnancy beta‐cell dysfunction and insulin resistance in women who develop GDM may compromise their capacity to adapt to the metabolic changes (i.e., increased insulin resistance) which predominantly occur in late pregnancy (Buchanan, 2001; Metzger et al., 2007). Additionally, various constituents of fruits, vegetables, and whole grains in a healthy diet (i.e., antioxidants, phytochemicals, and fiber) may decrease GDM risk through their reductive effects on oxidative stress and insulin demand (Hamer & Chida, 2007). The lower glycemic load of healthy diets in contrast with unhealthy dietary patterns may also provide another reason in support of our findings since the glycemic load is a strong predictor of postprandial glycemic response (Pustozerov et al., 2020).

According to the subgroup analysis, the results for healthy dietary patterns depend on study design, methods used to assess dietary patterns, and the time period of data collection (prepregnancy or during pregnancy). The nonsignificant association in cross‐sectional and case–control studies and in studies that collected dietary data during pregnancy might be related to behavioral changes after the diagnosis of GDM (Okely et al., 2019) that would result in favorable dietary changes. The null association between GDM and dietary patterns determined by a posteriori methods may be attributed to the variations in factor loading of food items which varied between studies. In addition, they may be predominantly different in food composition despite having some shared components.

Results of subgroup analysis based on adjustment for energy influenced both results for healthy and unhealthy eating patterns. While the associations were statistically significant in studies that assessed energy intake, no significant association was found in studies that did not. Indeed, variation in total energy intake due to body size, physical activity, and metabolic rate inevitably affects nutrient intake, and thereby obscure or even reverse the associations (Willett et al., 1997). The differences in geographical regions might be attributed to differences in living conditions, socioeconomic status, and other lifestyle factors that vary by country.

It is acknowledged that the analysis has some limitations which should be taken into consideration when interpreting the results. First, dietary intakes were examined through self‐reported tools which are prone to recall bias, although they are generally accepted. Furthermore, variations in the instruments used to assess dietary intakes can lead to a combination of measurement errors and misclassification of participants which consequently affect the results. Besides this, variations in GDM definitions between medical organizations can affect the generalizability of our findings and should be accounted for in medical implementation (Popova et al., 2016). Second, although it was tried to match the definition of healthy and unhealthy diets with the employed predefined criteria, differences in factor loadings of individual foods in a posteriori‐identified dietary patterns may result in misclassifications of dietary patterns. Third, since there is no cutoff level to determine adherence to a healthy or unhealthy diet, it is not possible to conclude how much adherence would be enough to effectively reduce GDM risk. Forth, although the most adjusted estimates were used in the analysis, because of the observational design of included studies, the effects of residual and unknown confounders cannot be completely ruled out.

The meta‐analysis also has several strengths. A comprehensive literature search was conducted, and in comparison with the previously published meta‐analysis, more relevant articles were identified and included in the analysis, and also a dose–response analysis was performed. The maximally adjusted models were enrolled in the analysis to consider the effect of various confounders, and various potential sources of heterogeneity were examined in the study. Moreover, the results of sensitivity analysis suggested that the results were robust, particularly for healthy dietary pattern. The lack of publication bias is another strength of this study.

## 5 CONCLUSION

The findings of this meta‐analysis support a strong inverse association between the healthy dietary pattern and GDM risk, whereas an unhealthy dietary pattern was associated with a tendency toward higher risk for GDM. However, the results are based on observational studies and limited numbers of prospective cohort studies which may not exactly allow inferring the causality, and there is a need for further prospective cohort studies and clinical trials to explore the causality between healthy and unhealthy dietary patterns and GDM.

## FUNDING INFORMATION

This study was supported by Isfahan University of Medical Sciences (IR.MUI.RESEARCH.REC.1398.800; Project Number: 198247).

## CONFLICT OF INTEREST

The authors declare that there are no conflicts of interest.

## ETHICS STATEMENT

This study does not involve any human or animal testing.

## Supporting information


Figure S1

Figure S2

Figure S3

Figure S4

## Data Availability

The data that support the findings of this study are available from the corresponding author upon reasonable request.
