# Supplementary figures and images for: Dose–response association between dietary patterns and gestational diabetes mellitus risk: A systematic review and meta‐analysis of observational studies
Source: Food Sci Nutr. 2022 Sep 27;11(1):57–92. doi: 10.1002/fsn3.3042 (PMC9834857; doi:10.1002/fsn3.3042)

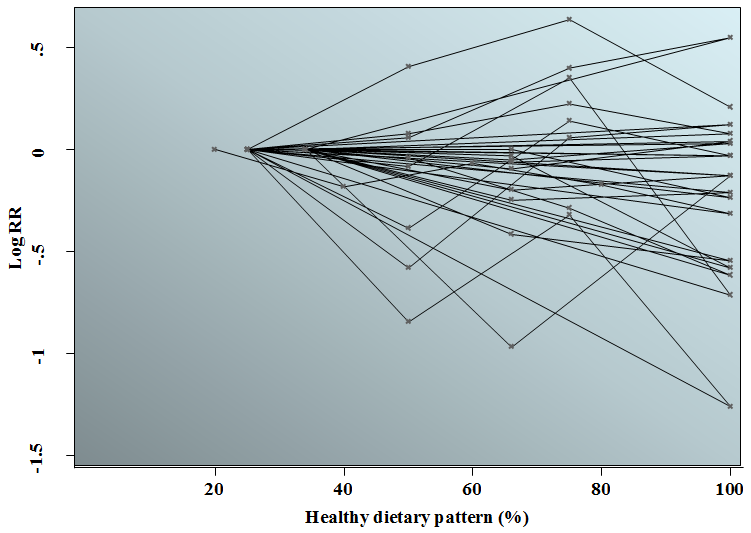

Supplement: Supplementary file 1 — Figure S1 [file FSN3-11-57-s002.tif]

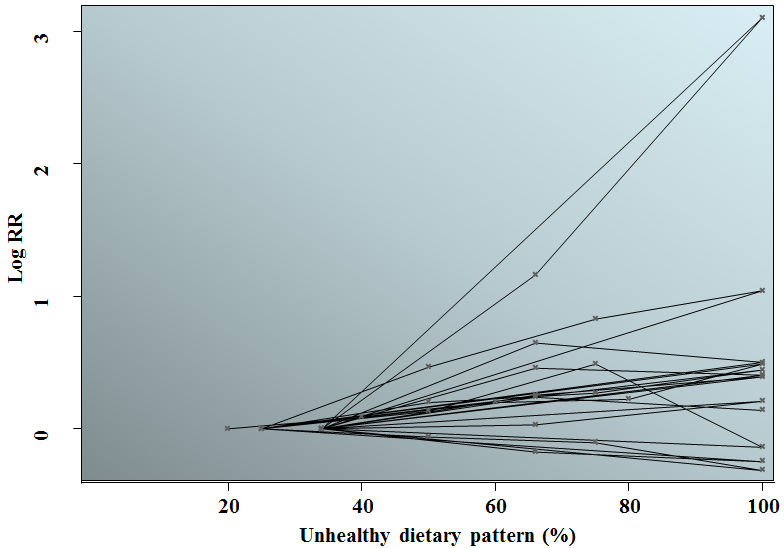

Supplement: Supplementary file 2 — Figure S2 [file FSN3-11-57-s003.tif]

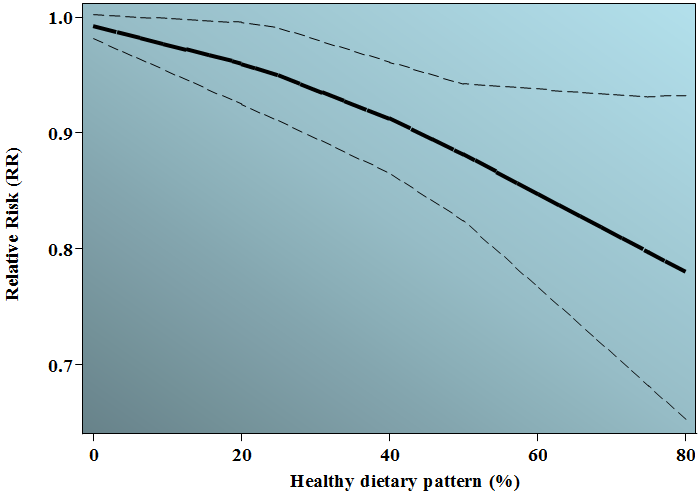

Supplement: Supplementary file 3 — Figure S3 [file FSN3-11-57-s004.tif]

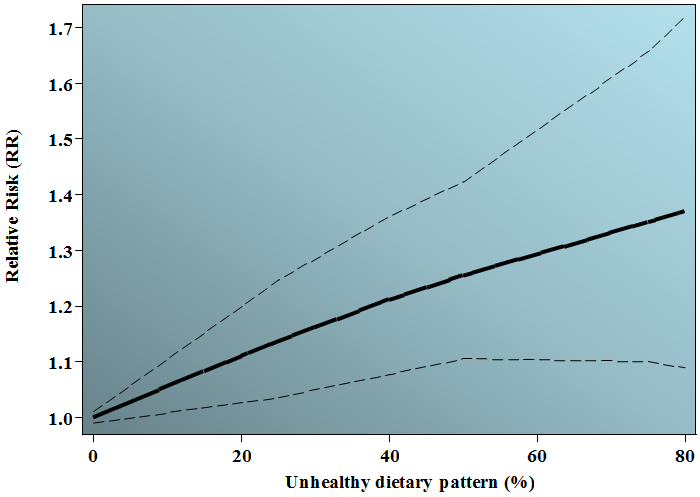

Supplement: Supplementary file 4 — Figure S4 [file FSN3-11-57-s001.tif]
